# Computational approaches to apply the String Edit Algorithm to create accurate visual scan paths

**DOI:** 10.16910/jemr.17.4.4

**Published:** 2024-11-15

**Authors:** Ricardo Palma Fraga, Ziho Kang

**Affiliations:** University of Oklahoma, USA

**Keywords:** eye movement, thresholds, algorithms, scan path, eye tracking, gaze

## Abstract

Eye movement detection algorithms (e.g., I-VT) require the selection of thresholds to identify eye fixations and saccadic movements from gaze data. The choice of threshold is important, as thresholds too low or large may fail to accurately identify eye fixations and saccades. An inaccurate threshold might also affect the resulting visual scan path, the time-ordered sequence of eye fixations and saccades, carried out by the participant. Commonly used approaches to evaluate threshold accuracy can be manually laborious, or require information about the expected visual scan paths of participants, which might not be available. To address this issue, we propose two different computational approaches, labeled as “between-participants comparisons” and “within-participants comparisons.” The approaches were evaluated using the open-source Gazebase dataset, which contained a bullseyetarget tracking task, where participants were instructed to follow the movements of a bullseye-target. The predetermined path of the bullseye-target enabled us to evaluate our proposed approaches against the expected visual scan path. The approaches identified threshold values (220°/s and 210°/s) that were 83% similar to the expected visual scan path, outperforming a 30°/s benchmark threshold (41.5%). These methods might assist researchers in identifying accurate threshold values for the IVT algorithm or potentially other eye movement detection algorithms.

## Introduction

Exploring how humans visually search their environment has been an important topic in eye-tracking research. One way to study how we visually explore the environment is to analyze visual scan paths, the time-ordered sequence of eye fixations and saccades (20), created throughout a task. Visual scan paths have been analyzed across various domains, such as healthcare (17; 8), air traffic control ( 27; 21; 34; 33), aircraft piloting (37; 29), automobile driving (19), deepwater horizon operations (36), education ( 41; 39), among others.

**Figure 1:**
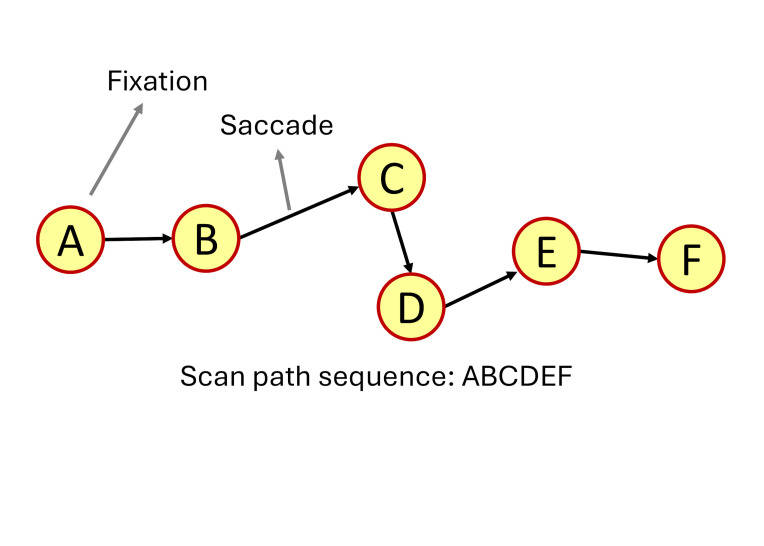
Representative examples of a visual scan path, highlighting one eye fixation and a saccadic movement. The scan path sequence created by the visual scan path is ABCDEF.

In order to create visual scan paths or scan path sequences (Figure 1), researchers apply eye movement detection algorithms, such as the Velocity-Threshold Identification algorithm (I-VT) (38), to identify eye fixations and saccades from the gaze data collected by an eye tracker. The I-VT algorithm classifies gaze samples collected by an eye tracking device as eye fixations or saccadic movements, based on a gaze velocity threshold. More specifically, if the gaze velocity between two gaze points is less than the gaze velocity threshold, the points are classified as belonging to an eye fixation, otherwise, it is considered to be a saccadic movement (38).

The choice of threshold value of the I-VT algorithm is important, as it might affect the resulting visual scanpath that can be created. Prior research has shown how the ability of eye movement detection algorithms to properly identify and classify eye fixations and saccades may be influenced by the threshold selected (38; 2). In the case of the I-VT algorithm, different gaze velocity thresholds can affect the number of eye fixations identified (23). Therefore, different visual scan paths might be created at different threshold values due to the varying number of eye fixations and saccadic movements identified across thresholds (45).

Furthermore, the threshold values capable of creating accurate visual scan paths might vary between studies. The accuracy of threshold values might be affected by differences in participant population, such as older individuals (5), the task to be completed and the stimuli presented (44), the device (e.g., mobile phone) in which participants complete tasks (42), among others. As a result, a gaze velocity threshold of 30 °/s might be accurate when investigating how syntax highlighting affects code comprehension (3), while a gaze velocity threshold of 60 °/s might be accurate when participants are tasked with reading text (26). In order to create accurate visual scan paths using the I-VT algorithm, researchers need to select appropriate threshold values for their respective study.

One way to identify an appropriate threshold value is to evaluate how the scan paths sequences created at multiple thresholds differ from an ideal scan path sequence (i.e., the scan path sequence actually carried out by the participant). Prior studies have successfully used this approach by calculating the string edit distance of scan path sequence created at a threshold value to the ideal scan path sequence (5; 16). The thresholds that resulted in scan path sequences that were the most similar to the ideal scan path sequence were considered to be accurate threshold values.

However, such an approach requires the researcher to know the likely eye movements that could take place ahead of time (i.e. an ideal visual scan path), which might not always be possible (40), or to manually create the ideal scan path sequence from the gaze data collected, which can be very time consuming (5). Consider the process of manually identifying the ideal scan path sequence of a participant whose eye movements were collected using, for example, the Tobii Pro Glasses II eye tracker, which collects gaze data every 10 milliseconds. For a short 5 second duration experiment, a researcher would need to manually process approximately 500 gaze points in order to define the ideal scan path sequence.

Furthermore, to the best of our knowledge, only one prior study has proposed an approach to identify appropriate thresholds when ideal visual scan path sequences are not available (4). More specifically, their approach consists of creating multiple variations of the Dispersion-Threshold Identification (I-DT) algorithm, where each variation uses a different definition of dispersion. To identify an accurate threshold, the scan path sequences created by one algorithm variation are then compared to the scan path sequences created by every other algorithm variation by calculating their string edit similarity. The threshold value at which all variations of the I-DT algorithm created similar scan path sequences are then considered to be the accurate thresholds. However, such an approach relies on the researcher’s subjective judgement to create multiple variations of the eye movement detection algorithm used in order to create and compare multiple scan path sequences. Furthermore, the I-DT algorithm is not readily available across commonly used eye tracking software, such as Tobii Pro Lab, which uses a version of the I-VT algorithm (30). Therefore, it might be challenging for some researchers to carry out the approach in their respective studies.

As a result, the present work expands upon these prior research efforts by introducing two computational approaches, between-participant and within-participant comparisons, to compare scan path sequences in order to identify and select accurate thresholds for the I-VT algorithm. In more detail, between-participants comparisons calculate the average similarity between the scan path sequences of multiple participants at the same threshold value. On the other hand, within-participant comparisons calculate the average similarity of the scan path sequence created at one threshold value to the scan path sequences created at every other threshold value for a single participant. To evaluate the ability of the two approaches to identify acceptable threshold values, we apply them to the open-source GazeBase dataset (15), which contains the eye movements of 322 participants instructed to follow the movements of a bullseye target changing locations on a computer display. In addition, the performance of the acceptable threshold values identified by each method are compared to a benchmark 30 °/s gaze velocity threshold, a threshold value recommended in some implementations of the I-VT algorithm (30).

The paper is structured as follows. The two methods are introduced and explained alongside worked-out examples. Afterwards, the methods are applied to the GazeBase (15) dataset. Lastly, the results are presented and interpreted in the discussion section, alongside limitations and avenues of future research.

## Proposed algorithms

### String edit similarity of scan path sequences as a measure to evaluate and select thresholds

When selecting the gaze velocity threshold to identify eye fixations and saccadic movements using the I-VT algorithm, a researcher may inadvertently select a gaze velocity threshold that may be too low or too high (30, 42).

**Figure 2:**
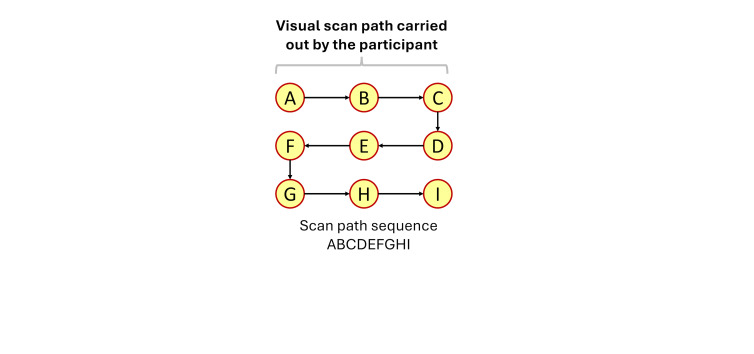

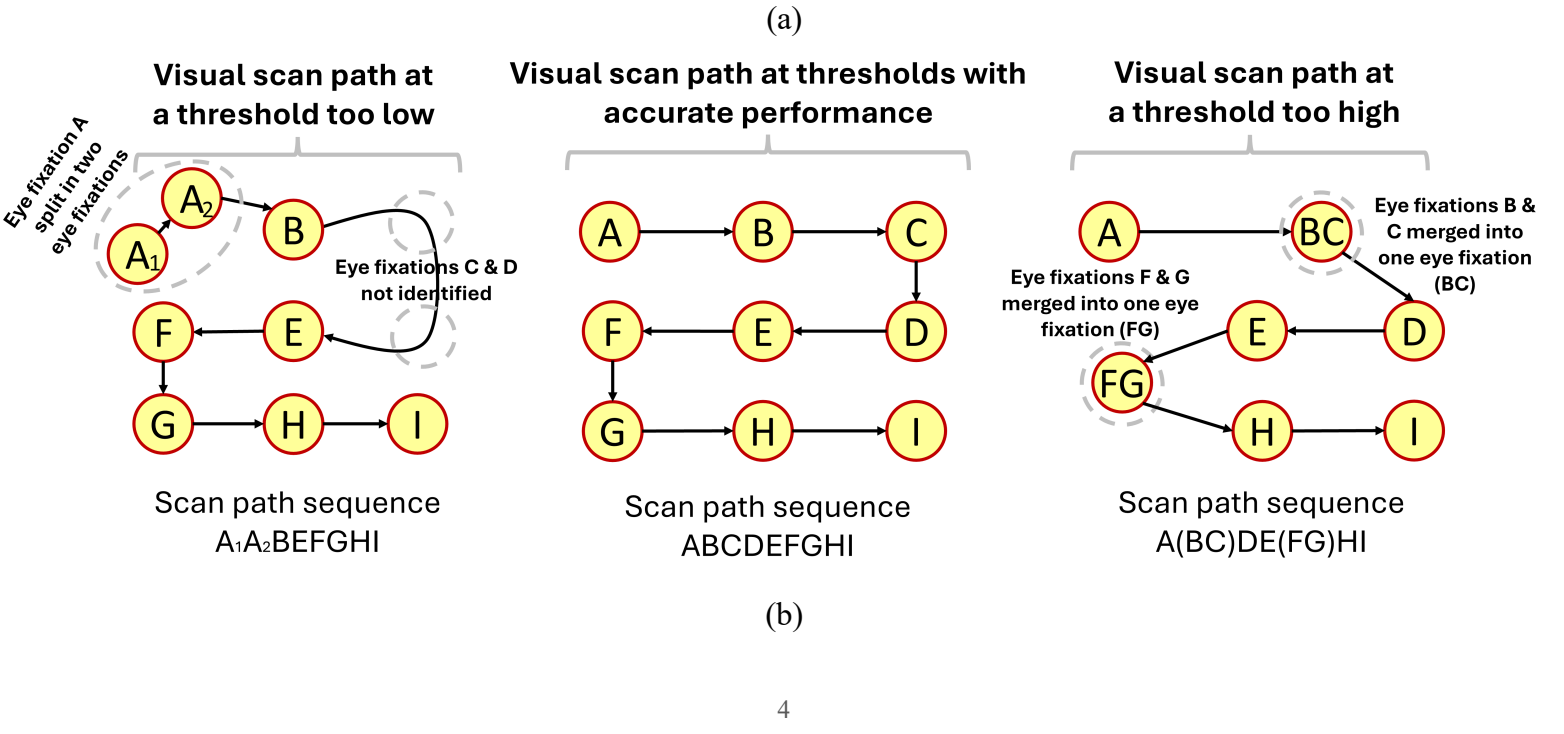
Simplified example showcasing the impact of thresholds values on scan paths sequences. Note: Figure 2(a) contains the visual scan path (and the scan path sequence) of a participant instructed to follow a dot (i.e., stimuli) moving in an ideal manner back and forth across the display; Figure 2(b) showcases simple examples of visual scan paths (and the scan path sequences) created at thresholds too low, acceptable, and too high.

When the gaze velocity threshold is too low, gaze samples belonging to eye fixations can be misclassified as saccadic movements, which can lead to an eye fixation being erroneously split into separate eye fixations (38). In some cases, a threshold too low might even fail to identify that an eye fixation took place at all, as all the gaze samples belonging to that eye fixations are classified as belonging to a saccadic movement. Consider the example presented in Figure 2. Using a threshold too low (Figure 2(b), left) identified the eye fixations B, E, F, G, H, and I carried out by the participant. However, eye fixation A was split into two eye fixations, while eye fixations C and D were not identified at all. As a result, the scan path sequence created with a threshold contains the split eye fixation and does not contain the two missing eye fixations. In addition, it also indicates that the participant carried out eye movement transitions that never took place, such as eye fixation B followed by eye fixation E.

On the other hand, when the gaze velocity threshold is too high, gaze samples belonging to saccadic movements might be misclassified as belonging to an eye fixation. In such cases, it might be possible that two eye fixations separated by a saccadic movement could potentially be combined into a singular eye fixation. Furthermore, the combined eye fixation may also be placed at a location that the participant never actually observed (4). Consider the example provided in Figure 2. A threshold too high (Figure 2(b), right) combined multiple eye fixations together, such as eye fixations B and C into a single eye fixation (BC), as well as eye fixations F and G into a single eye fixation (FG). Lastly, the combination of multiple eye fixations impacted the location the eye fixations (BC) and (FG), placing them at a location that the participant never observed.

However, somewhere in between the thresholds that are too low or too high exists a range of thresholds that lead to acceptable performance – those threshold values can accurately identify the eye fixations and saccadic movements carried out by participants, as can be observed in Figure 2(b, center).

**Figure 3:**
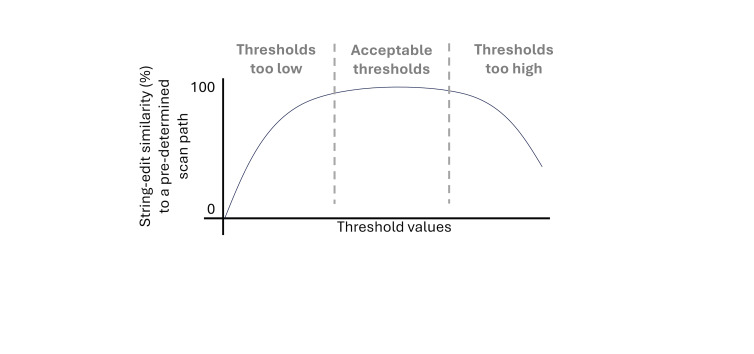
Example visualization of the relationship between threshold values and string edit similarity to an ideal scan path sequence.

As mentioned previously, one potential approach to determine whether a threshold may be too low, acceptable, or too high is to systematically compare scan path sequences created at each threshold to an ideal scan path sequence based on their string edit distance (5; 16). The string edit distance between two scan path sequences is defined as the number of insertions, deletions, and substitutions needed to convert one scan path sequence into the other sequence, normalized by the length of the largest sequence (35). The string edit distance can be converted into a similarity measure by subtracting 1 from the distance value (35). Prior studies that have carried out this systematic approach, comparing visual scan path sequences to an ideal scan path sequence (5; 16), have showcased that the relationship between threshold values and the string edit distance to an ideal scan path sequence, depicted in Figure 3 as the equivalent similarity value, appears to increase at low thresholds, remain stable for a range of thresholds, after which it begins to decrease.

However, in many instances, an ideal scan path sequence is not readily available for researchers to compare with in order to evaluate thresholds. To address this challenge, the following section introduces two methods to approximate the relationship between thresholds and string edit similarity to facilitate the selection of accurate threshold values.

### Between-participant comparisons

Under controlled experimental conditions, such as those containing participants with similar characteristics, such as expertise (43), given the same task and set of instructions ( 9; 6), participants might carry out scan path sequences that are very similar to each other. Under such circumstances, it might be possible to attribute some differences in scan path sequences between participants to the thresholds values selected. Accurate threshold values might lead to higher similarities between scan path sequences of participants, while thresholds too low or too high might have lower similarities due to missing eye fixations, split eye fixations, as well as the presence of unlikely transitions in scan path sequences.

Leveraging this assumption, we adapt the between-participant comparisons similarity metric, commonly used in prior eye tracking research (11, 1), to evaluate the string edit similarity between participants’ scan path sequences. In our implementation, the between-participant comparisons similarity is calculated across all threshold values explored, rather than at a singular fixed threshold as done in prior studies.

The calculation of between-participant comparisons similarity at each threshold is as follows. Let $X_{i}=\left\{x_{1},x_{2},\ldots ,x_{j}\;\right|\;1\;\leq j\leq p,\;j\in \mathbb{N}\}$, where $X_{i}$ contains the set of scan path sequences at the ith threshold for all p participants and $x_{j}$ represents the scan path sequence of the jth participant. From the set $X_{i}$, one can define all m two-scan path sequence combinations between participants at the ith threshold as $W_{i}=\left(\begin{array}{c} X_{i}\\ 2 \end{array}\right)=\left\{\left(x_{j},x_{k}\right)\right|\;1\leq j<k\leq p,\;j\in \mathbb{N},k\in \mathbb{N}\}$. For all combinations, the average string edit similarity ${\bar{h}}_{i}$ at the threshold ith between all participants can then be calculated using equation (3).

(3) $${\bar{h}}_{i}=\frac{1}{m}\sum_{\left(x_{j},x_{k}\right)\;\in \;W_{i}}^{}S\left(x_{j},x_{k}\right)\;\;\;\;\forall \;i\in \{1,2\ldots ,t\}$$

**Figure 4:**
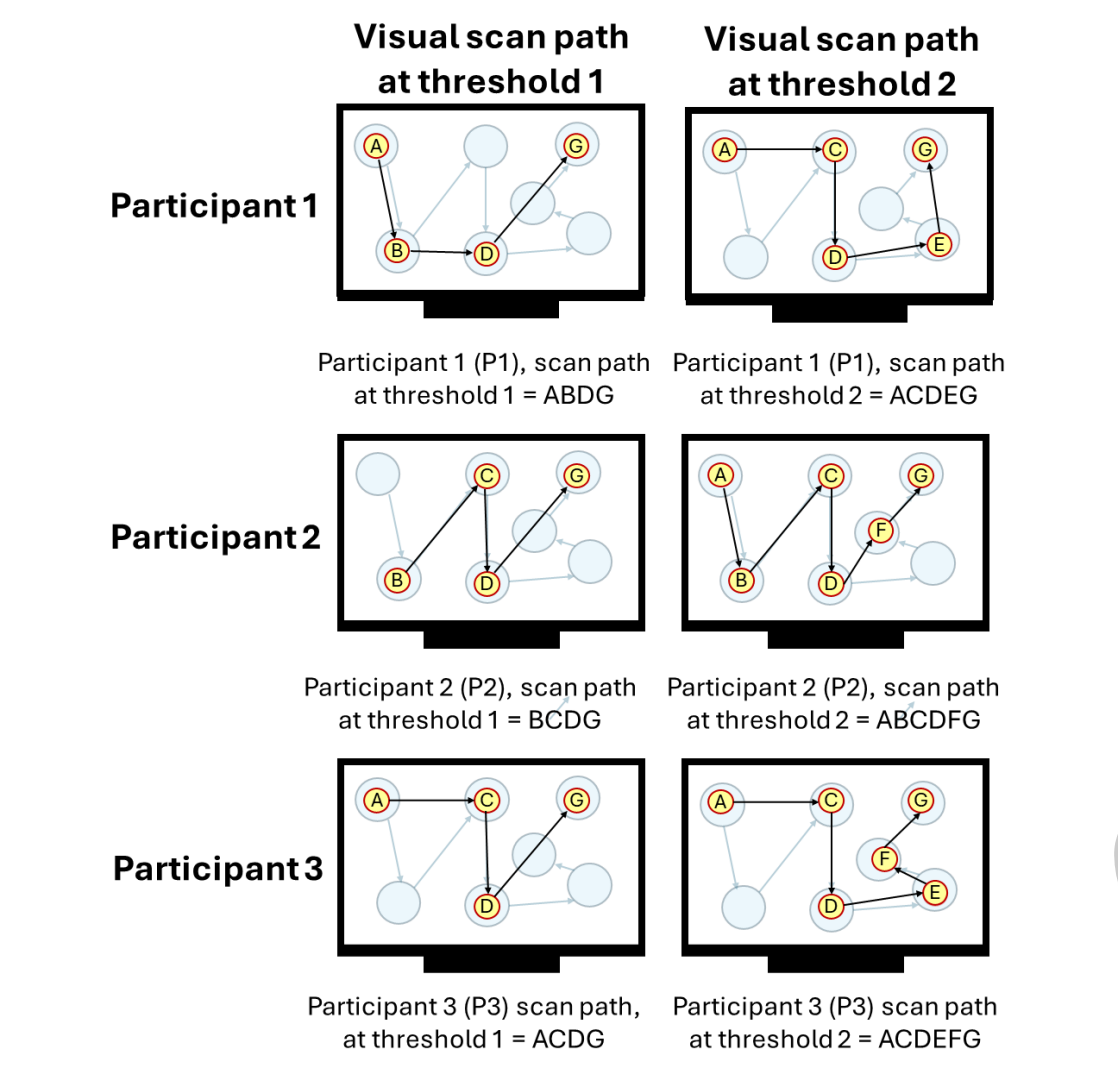

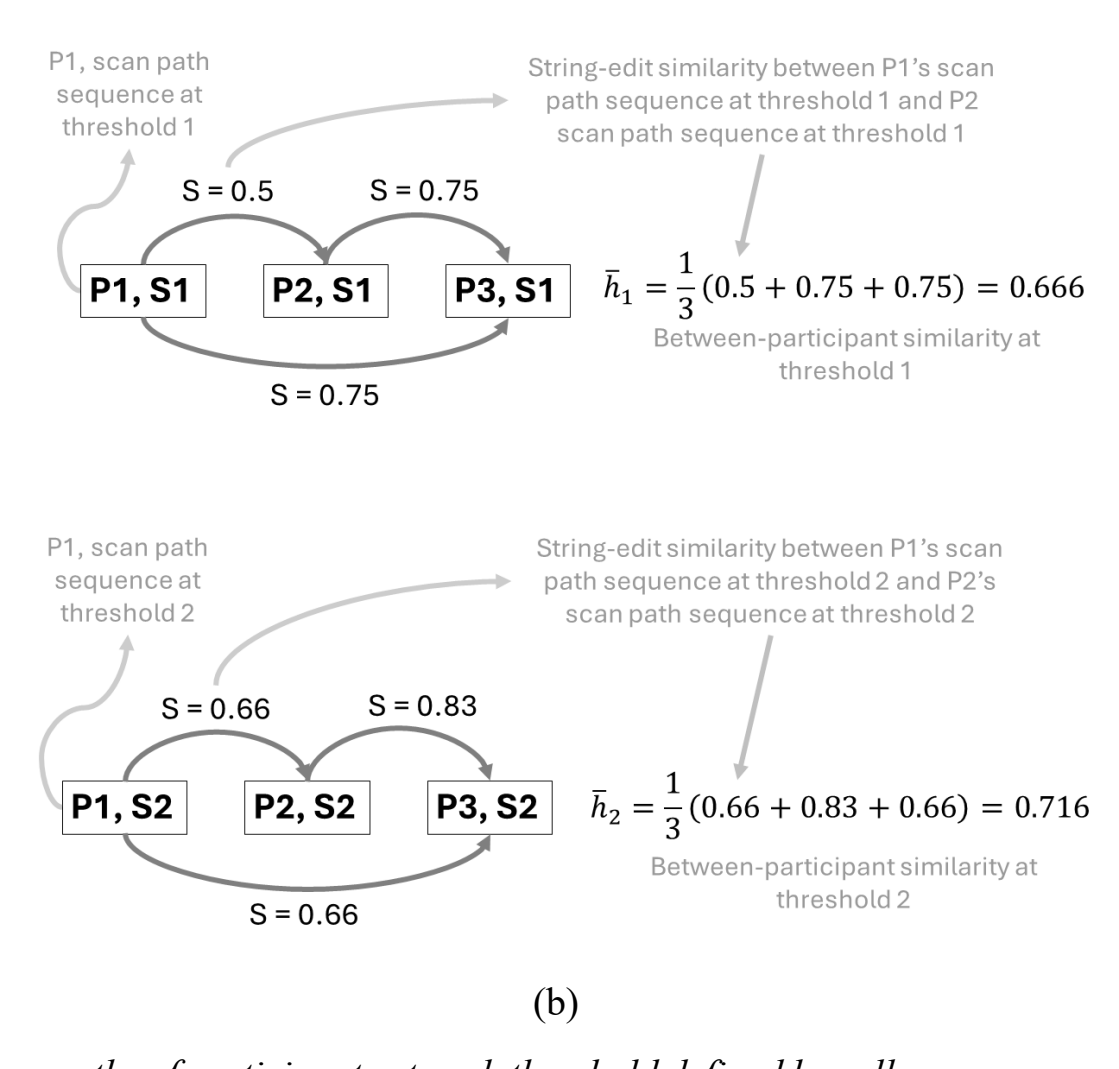
Example of between-participant comparisons calculations for three participants who were instructed to follow the ideal movement of a blue dot on a display. Note: Figure 4(a) contains the visual scan paths of participants at each threshold defined by yellow circles (i.e., eye fixations) indexed in alphabetical order connected with black arrows (i.e., the saccadic movements). The movement of the blue dot is denoted by transparent blue circles connected with blue arrows. The letter assigned to each eye fixation is based upon whether the eye fixation took place within the corresponding blue dot. There are a total of 3 combinations possible between participants at each threshold (i.e., (P1, P2), (P1, P3), and (P2,P3)). Figure 4(b) showcases the calculations of the average string edit similarity between participants at the ith threshold using equation (3).

Figure 4(a) showcases an example of the between-participant comparisons similarity calculated for the scanpath sequences created at two thresholds (1 and 2) carried out by three participants (P1, P2, and P3). Equation (3) is applied in Figure 4(b) to compare all possible combinations of two-scan path sequences among participants. The results indicate that the threshold with the highest average string edit similarity between participants would be threshold 2 (0.716 at threshold 2 vs 0.666 at threshold 1).

### Within-participant comparisons

Another way to identify accurate thresholds might be to compare the scan path sequences created at one threshold to the scan path sequences created at every other threshold.

Scan path sequences created at a threshold too low or too high might have a lower similarity to a scan path sequence created at an accurate threshold. As mentioned previously, scan path sequences created at threshold too low or too high might have missing eye fixations, contain unlikely transitions between eye fixations, or even split one eye fixation into multiple eye fixations. On the other hand, scan path sequences created at an accurate threshold might contain fewer errors (e.g. missing eye fixations) or none at all. Therefore, one might expect that the similarity between the scan path sequences created at an accurate threshold, and one created at threshold too low or too high, ought to be lower than the similarity between scan path sequences created at accurate thresholds.

Based on this assumption, we propose the within-participant comparisons similarity metric to calculate the average string edit similarity of the scan path sequence of one threshold to the scan path sequences created at every other threshold. Comparing the scan path sequence created at a threshold to the scan path sequences created at every other threshold might serve as a way to approximate the relationship shown in Figure 3.

The calculation of the proposed within-participant comparisons similarity for a single participant is as follows. Let $F=\left\{f_{1},f_{2},\ldots ,f_{i}\;\right|\;1\;\leq i\leq t,\;i\in \mathbb{N}\}$ represent the set of scan paths sequences created at each threshold, where $f_{i}$ represents the scan path sequence created at the ith threshold, and t the total number of thresholds. The average string edit similarity $v_{i}$ of the scanpath sequence at threshold i to the scan path sequence at every other threshold can be calculated using equation (4).

(4) $$v_{i}=\frac{1}{t-1}\sum_{j=1;i\neq j}^{t}S\left(f_{i},f_{j}\right)\;\;\;\;\;\;\;\forall \;i\in \{1,2\ldots ,t\}$$

Where S represents the string edit similarity function (explained in detailed below in the methods section), $f_{i}$ and $f_{j}$ the scan path sequences at thresholds i and j in the set F. Note that the average is calculated by dividing t−1, instead of simply t, as there is a total of t−1 similarity calculations. The similarity between one scan path sequence to itself is never calculated (i.e., i≠j).

In addition, when considering multiple participants, the outputs of equation (1) can be averaged across participants to identify the threshold that creates the most similar scan path sequence for all participants. Let $V=\left\{v_{ij}\;\right|\;1\leq i\leq t,1\leq j\leq p,\;i\in \mathbb{N},\;j\in \mathbb{N}\}$ contain the set V of average string edit similarity $v_{ij}$ at the ith threshold for the jth participant, where p represents the total number of participants. Afterwards, the average string edit similarity ${\bar{v}}_{i}$ at threshold i across participants can be calculated using equation (5).

(5) $${\bar{v}}_{i}=\frac{1}{p}\sum_{j=1}^{p}v_{ij}\;\;\;\;\;\;\;\forall \;i\in \{1,2\ldots ,t\}$$

**Figure 5:**
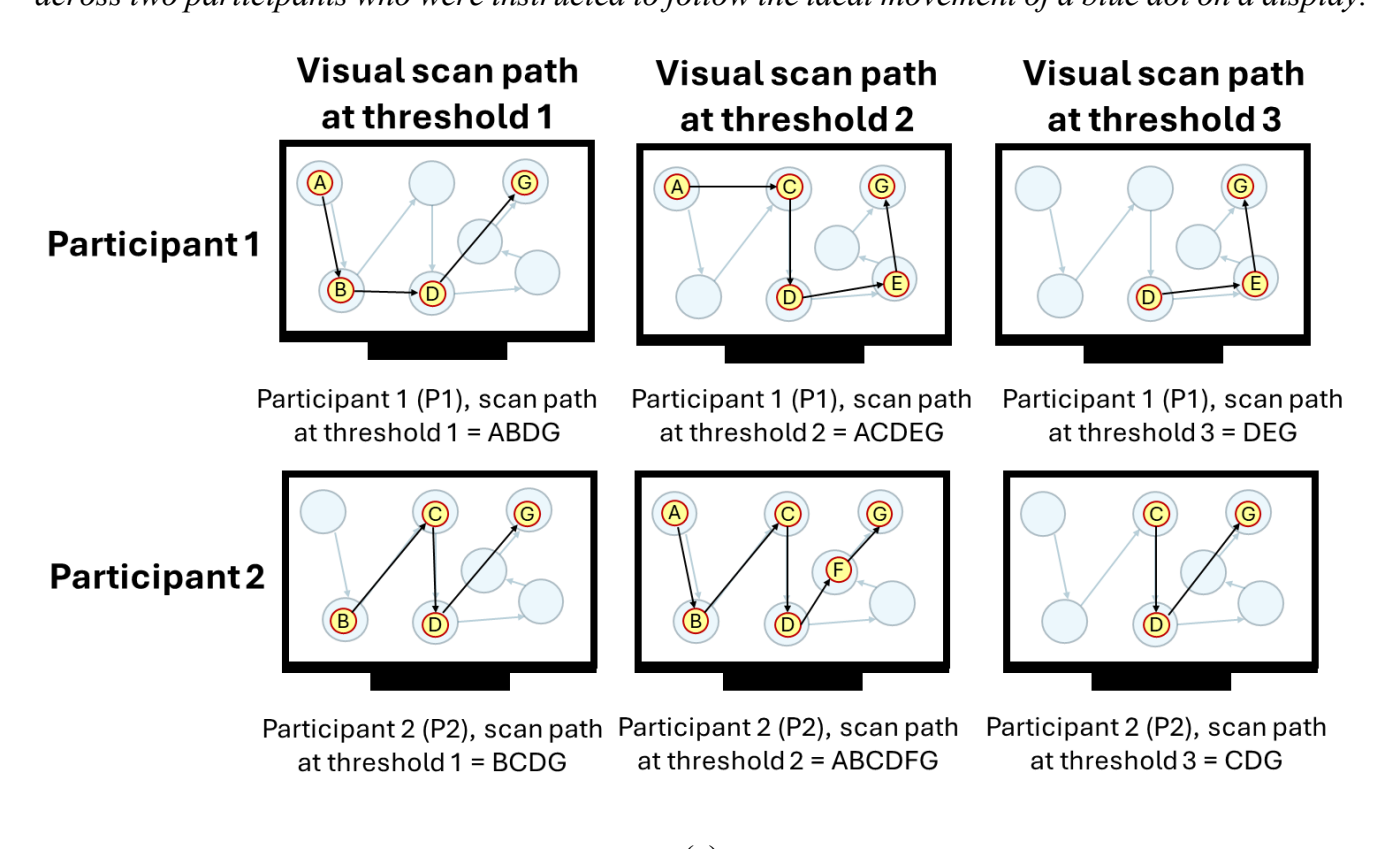

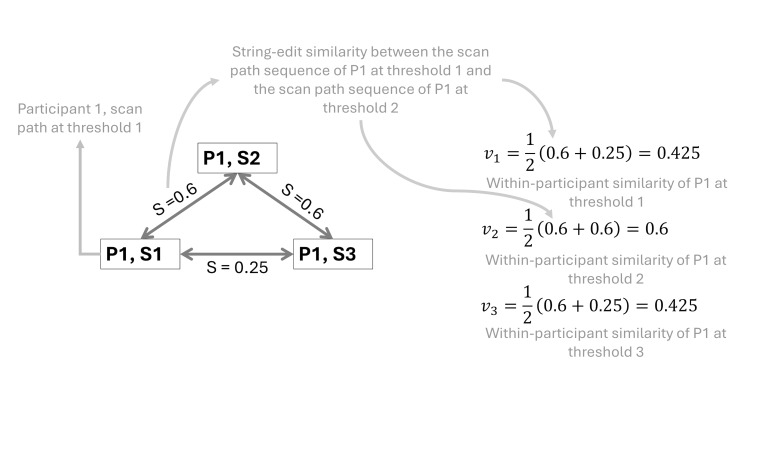

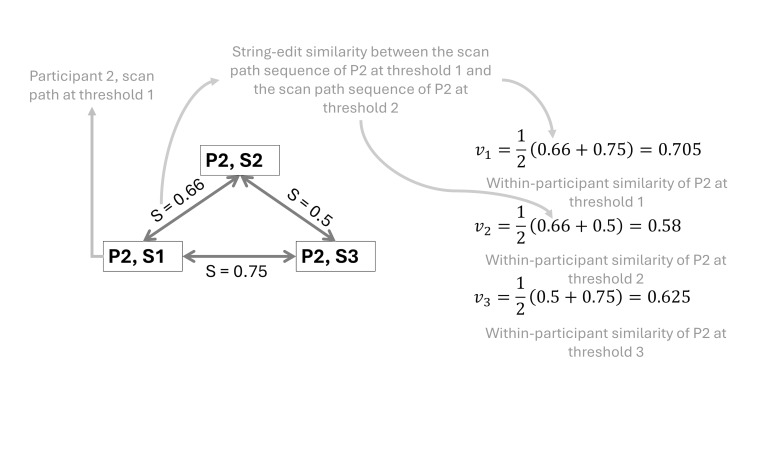

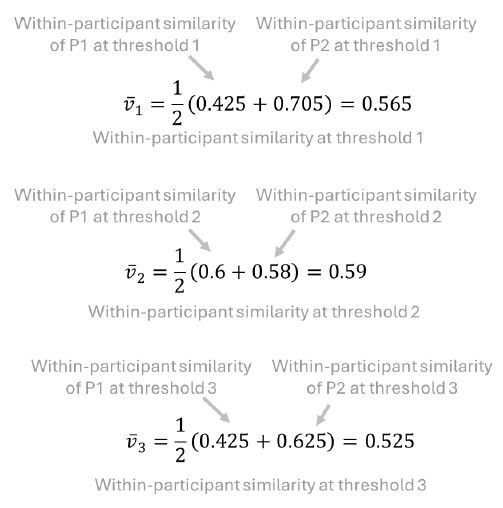
Representative example of the calculation of the average string edit similarity at each threshold across two participants who were instructed to follow the ideal movement of a blue dot on a display. Note: Figure 5(a) contains the visual scan paths of participants at each threshold defined by yellow circles (i.e., eye fixations) indexed in alphabetical order connected with black arrows (i.e., the saccadic movements). The movement of the blue dot is denoted by transparent blue circles connected with blue arrows. The letter assigned to each eye fixation is based upon whether the eye fixation took place within the corresponding blue dot. Figure 5(b and c) showcases the calculations of the average string edit similarity at the iththreshold for each participant using equation (4). Figure 5(d) contains the calculation of the average string edit similarity at each threshold across two participants using equation (5).

Figure 5(a) shows an example of the within-participant comparisons similarity being calculated for the scan path sequences at three thresholds (1, 2, and 3) carried out by two participants (P1 and P2). Equation (4) is applied in Figure 5(b and c) to calculate the threshold that creates the scan path sequence most similar to the scan path sequences at every other threshold for each participant. Lastly, equation (5) is applied in Figure 5(d), showcasing that the threshold with the highest average similarity to every other scan path sequence among the two participants would be threshold 2 (0.59 at threshold 2 vs 0.565 at threshold 1 and 0.525 at threshold 3).

## Evaluation

The two approaches proposed in the previous chapter were applied to the Random Saccade (RAN) task in the GazeBase dataset (15). The task consisted of participants fixating and following the movement of a bullseye target that changed locations throughout a computer display. The GazeBase dataset and the RAN task were selected as an ideal scan path sequence is readily available for each participant (i.e., the movement of the bullseye target participants were instructed to follow), allowing us to evaluate the performance of both methods, as we assume that we do not know what the ideal scan path sequence is.

In this section, we first briefly introduce key elements of the Gazebase dataset, summarized from the original work of Griffith et al (15), to facilitate comprehension of the present work. For additional details regarding Gazebase, we encourage readers to visit their original work (15). Afterwards, the steps taken to process the eye movement data are explained, including defining the ideal scan path sequences and the calculation of string edit similarity. Lastly, the data analysis procedure is described.

### A. Gazebase dataset

**Figure 6:**
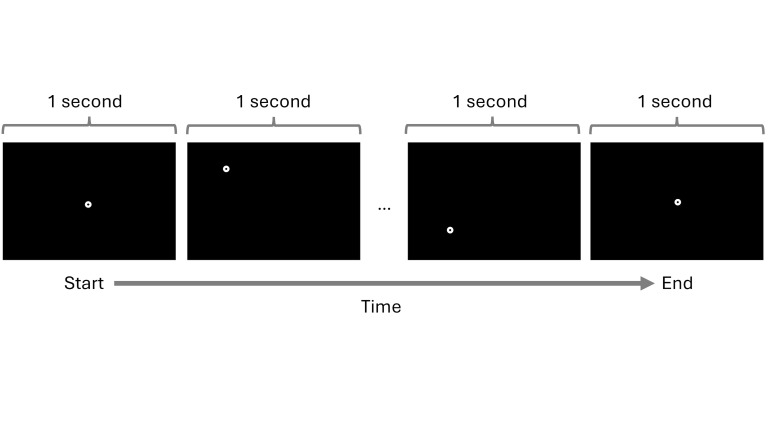
Example visualization of the RAN task carried out by participants in the GazeBase dataset. Note: A total of 100 bullseye targets were shown to participants. The location of the targets was different for each participant. This image was created based on the example included in the GazeBase dataset.

A total of 322 college students (171 self-identifying as male, 151 as female; average 21.99 years of age, *SD*: 4.22) at Texas State University completed the RAN task. A representative visualization of the task can be observed in Figure 6. Participants were instructed to fixate and follow a bullseye target. Participants were shown 100 bullseye targets, each appearing on the display for 1 second, after which it would disappear and re-appear on another location on the display. The locations bullseye targets appeared were randomly selected across participants, with the exception of the starting and ending locations, which were both at the center of the display.

During the task, monocular eye movements of the left eye were collected using the EyeLink 1000 at a sample rate of 1000 Hz (SR Research, Ottawa, Ontario, Canada). Participants were seated 550 mm away from a 1680 x 1050 pixels (474 x 297 mm) computer monitor. The participants’ heads were stabilized using a chin and forehead rest. The eye tracker was calibrated to the participants eye movements following a 9-point calibration procedure. Afterwards, a validation procedure was conducted to ensure the accuracy of the eye movement data. The collected gaze samples and target bullseye positions were converted to degrees of visual angle (dva) based on the recording set-up (e.g., distance the participant was seated from the computer).

### B. Processing eye movement data

Participants with more than 10% of gaze samples missing, which may be missing as a result of blinking or partial occlusions of the eye as described by the authors of GazeBase (15), were removed from the analysis. Only 10 participants met the more than 10% missing samples criteria. As a result, only eye movements from 312 out of 322 participants were included in the final analysis.

The I-VT algorithm was applied to identify eye fixations and saccadic movements from the participant’s eye movements. A minimum eye fixation duration of 60 milliseconds (ms) was used, the default value in the Tobii implementation of the I-VT algorithm (e.g., 30). As the goal of the present work was to identify the range of acceptable gaze velocity thresholds, the I-VT algorithm was applied using a range of gaze velocity thresholds between 10 °/s and 400 °/s in increments of 10 °/s. Prior studies investigating the impact of the gaze velocity threshold have used similar threshold ranges. For instance, Larsson (25) used a range between 0 °/s and 400 °/s while Komogortsev et al. (23) used a range between 5 °/s and 300 °/s.

Although the I-VT algorithm identifies eye fixations and saccadic movements, additional processing is needed to determine whether an eye fixation took place on a bullseye target at the time it appeared on the screen. To achieve this, areas of interest (AOIs) were created for each bullseye-target (resulting in a total of 100 AOIs) and eye fixations were mapped to AOIs. The mapping process consisted of aligning eye fixations and AOIs temporally (i.e., if the eye fixation took place during the time a particular AOI was visible) and spatially (i.e., if the eye fixation took place on the AOI) (e.g., 22). The shape of each AOI was defined as a circle to match the circular shape of the bullseye targets, with a radius of 2 dva. The radius of 2 dva was selected to account for the maximum validation error as described in GazeBase (15), with the exception of outliers. The validation error was defined as the Euclidean distance between the position the participant was instructed to look at and the position the eye tracker reported they were looking at.

To create the scan path sequences for each participant, AOIs were included in the participant’s scan path sequence if the mapping procedure indicated that an eye fixation took place within the AOI. Consider the scan path sequence of participant 1 at threshold A shown in Figure 4(a). In this example, one eye fixation was determined to have taken place in AOIs A, B, D, and G (denoted by the blue AOI at the location of the stimuli). As such, they are included in the scan path sequence of the participant in the order they were fixated on (i.e. ABDG). However, other AOIs that appeared in the environment are not included, as no eye fixations appeared to have taken place on the AOI at that gaze velocity threshold value.

In addition, multiple consecutive eye fixations on the same AOI were reflected in the participant’s visual scan path sequence. If a participant appears to fixate on AOI A two consecutive times, such as when an eye fixation on an AOI is erroneously split into two, the scan path sequence would include the AOI A twice (i.e., AA) rather than only once (i.e., A). We note this distinction as a pre-processing step when creating scan path sequences in some eye tracking studies is to group consecutive eye fixations on the same AOI together (e.g. 14; 12). Furthermore, note that in the current study, due to the presence of 100 AOIs, the AOIs were represented by double digit names (i.e., 00 for the first bullseye target, 01 for the second bullseye target, etc.). Lastly, the ideal visual scan paths were created based on the order the bullseye targets participants were instructed to follow appeared. More specifically, the ideal sequence created for each participant was: 00, 01, 02, … 98, 99 (separated by commas for legibility).

Finally, to calculate the string edit similarity between the ideal scan path sequences and the scan path sequences, the approach described in Privitera & Stark (35) was used. Here, the total number of insertions, deletions, and substitutions needed to convert one string into another was divided by the length of the longest sequence to calculate the string edit distance, which was then subtracted by 1 to calculate the similarity. However, given the high number of AOIs defined in the study, and their two-digit naming convention, the insertion, deletions, and substitutions were carried out at the two-digit level. Consider the following example scan path sequences containing two-digit AOIs: 01,02,03,04,05 (separated by commas for legibility) and 03,04,05. To convert the latter scan path sequence to the former, two AOIs must be inserted into the scan path sequence: 01 and 02. Thus, the string edit similarity between these two scan path sequences would be calculated as $1-\left(\frac{2}{5}\right)=0.6$. In other words, the two scan paths sequences are 60% similar to each other.

### C. Data analysis

The between-participants and within-participants comparisons were applied for all participants across multiple gaze velocity thresholds ranging from 10 °/s and 400 °/s in increments of 10 °/s.

In addition, the string edit similarity of the scan path sequences at each gaze velocity threshold to the ideal scan path sequence participants were instructed to follow was calculated. Gaze velocity thresholds that create scan path sequences with higher string edit similarity to the ideal scan path sequence are considered to be more accurate. In other words, said gaze velocity thresholds create visual scan paths that more accurately represent the eye movements participants were instructed to carry out (i.e. follow the movements of a target on a computer screen). As a result, the ‘ideal’ string-edit similarity values serves as a benchmark that can be used to compare the string-edit similarity values calculated by applying the between and within comparison methods.

Spearman’s rank correlations ( $r_{s}$) were calculated to evaluate the strength of the association between the string edit similarity values at each proposed method, and the string edit similarity values to the ideal scan path sequence. A significance level of *α* = 0*.*05 was used for the statistical test to evaluate whether the correlations were statistically significant.

Accurate gaze velocity thresholds were identified by visually observing the plots created for the two methods and the ideal scan path sequences. More specifically, the thresholds were identified by visually observing a high and stable region of similarity values, which both researchers agreed upon. The upper and lower bounds of the range of acceptable thresholds identified by each method were compared to those found for the ideal scan path sequence.

Lastly, the gaze velocity threshold values at which the highest string edit similarity occurs when applying within-participant and between-participant comparisons were identified. The accuracy of these gaze velocity threshold values was compared to the accuracy of a 30 °/s benchmark threshold value, a default value used in some implementations of the I-VT algorithm (30). A potential threshold value a researcher might find and use in their implementation of the I-VT algorithm without verifying its accuracy, an issue highlighted in prior eye tracking research (24; 32).

## Results

**Figure 7:**
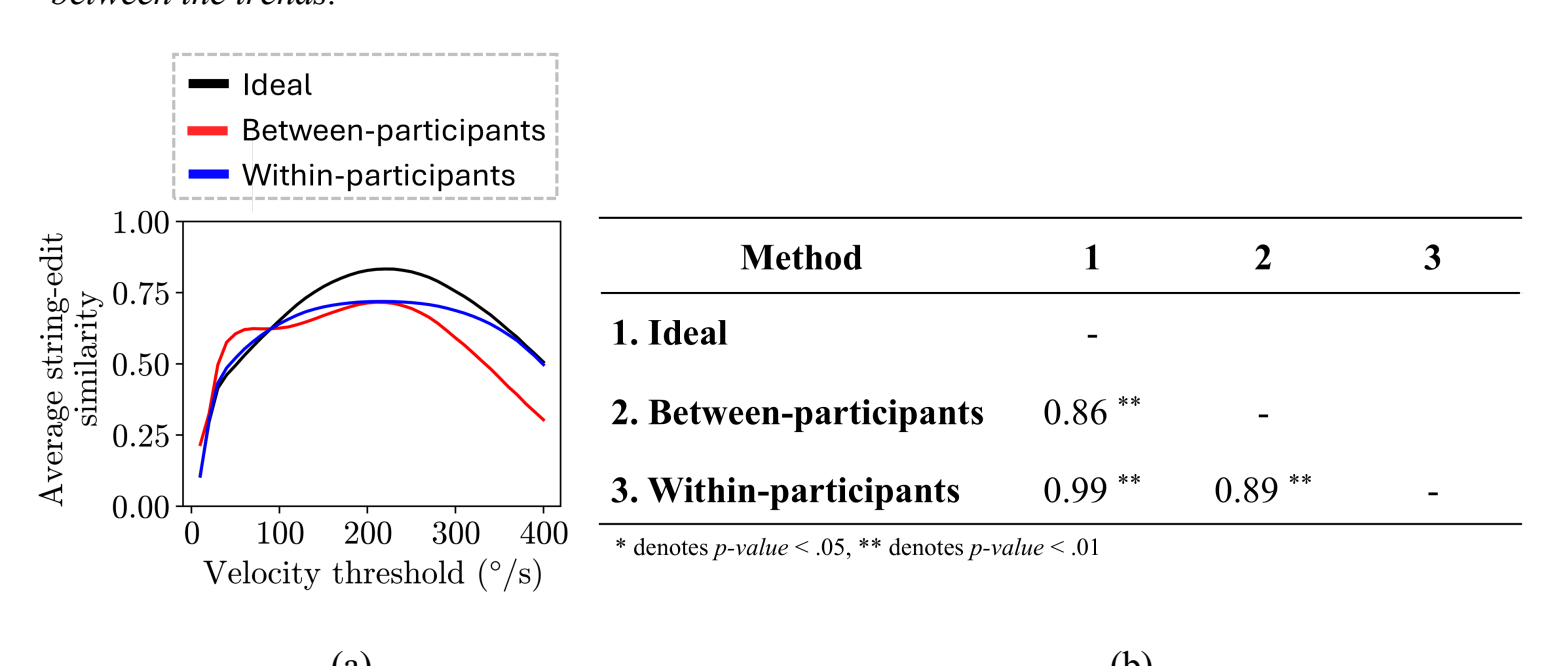
Plot of the string edit similarity over the range of thresholds evaluated (10 °/s to 400 °/s) for the between-participant comparisons (red line), within-participant comparisons (blue line), and the ideal scan path sequence string edit similarities (black line), as well as the Spearman correlations between the trends.

The average string edit similarity values for between-participants comparisons ( $r_{s}$ = 0.86, p-value < 0.01) and within-participants comparisons ( $r_{s}$ = 0.99, p-value < 0.01) were highly correlated with the ideal similarity values (Figure 7(b)). In addition, the average string edit similarity values of both methods were highly correlated with each other ( $r_{s}$ = 0.89, p-value < 0.01).

The within-participant comparisons similarity and the ideal similarity values followed similar trends across threshold values (Figure 7(a)). In more detail, the similarity values continuously increase until a maximum value is reached, after which the similarity values begin to decrease. Both trends reached a maximum similarity value (0.719 for within-participant comparisons similarity and 0.833 for ideal similarity) at the same gaze velocity threshold (220 °/s).

On the other hand, the between-participant comparisons similarity and the ideal similarity values showcased slightly different trends. More specifically, the between-participant comparisons trend contains a set of thresholds (60 °/s to 100 °/s) where the similarity values increase at a very small rate (an average increase of 0.00186 at each threshold), which is not present in the ideal similarity trend nor in the within-participant comparisons similarity trend. Afterwards, the between-participant comparisons similarity values continuously increase until a maximum similarity value (0.716) is reached at 210 °/s, close to the threshold value (220 °/s) at which the ideal similarity trend reached its maximum value.

**Figure 8:**
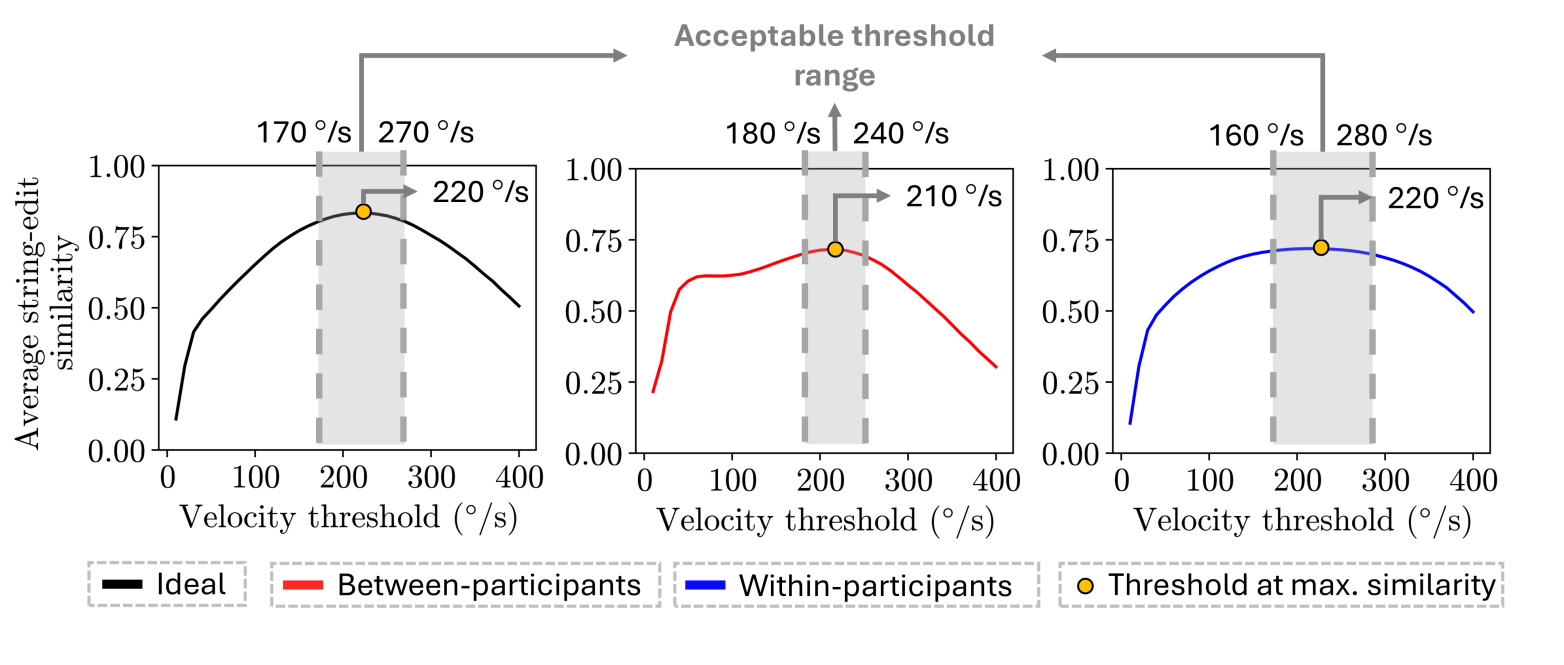
Plot of the string edit similarity over the range of thresholds values evaluated (10 °/s to 400 °/s) for each approach. The accurate threshold ranges identified for each approach are highlighted in gray, while the threshold at which the maximum similarity value occurs is denoted as a yellow circle.

The accurate gaze velocity threshold values identified using between-participant and within-participant comparisons largely overlapped with the threshold values identified when comparing to the ideal scan path sequence (Figure 8). The threshold range selected for the between-participant comparisons ranged from 180 °/s to 240 °/s, while the threshold range for the within-participant comparisons ranged from 160 °/s to 280 °/s, with both ranges having an average similarity value of 0.70 or higher. The range of thresholds for both methods largely agreed with the thresholds identified for the ideal similarity values, which ranged from 170 °/s to 270 °/s, with an average similarity of 0.80 or higher.

The gaze velocity threshold values identified by applying between-participant and within-participant comparisons had a string-edit similarity value of 0.78 or higher on the ideal trend (Table 1). In more detail, the thresholds range identified by the within-participant had 0.787 (160 °/s) and 0.789 (280 °/s) average similarity scores in the ideal trend. For the thresholds range identified via the between-participants method, the average similarity scores in the ideal trend were 0.812 (180 °/s) and 0.828 (240 °/s).

Lastly, the gaze velocity threshold values with the highest average string edit similarity identified using the within-participant and between-participant comparisons outperformed the 30 °/s benchmark threshold. More specifically, the 210 °/s and 220 °/s threshold values identified by applying the between-participant and within-participant comparisons had an average string edit similarity of 0.831 and 0.833 on the ideal trend, respectively. On the other hand, the 30 °/s benchmark threshold had an average string edit similarity of 0.415 on the ideal trend.

## Discussion

We were able to identify and select gaze velocity threshold values for the I-VT algorithm to accurately classify eye fixations and saccadic movements for a bullseye-target tracking task. More specifically, two computational approaches, within-participants and between-participants comparisons, were introduced and applied to identify the accurate gaze velocity threshold values without using information regarding the ideal visual scan paths of participants (i.e. the movements of the bullseye target they were instructed to follow). The contribution of these two computational approaches might help other researchers to select gaze velocity threshold values for the I-VT algorithm to accurately identify eye fixations and saccadic movements in their respective applications.

### A. Approximating scan path sequence similarity to select accurate thresholds

The within-participants and between-participants approaches were capable of approximating the trend between thresholds values and ideal scan path sequence similarity values. More specifically, statistically high Spearman rank correlations were observed for both the within-participant ( $r_{s}$ = 0.99) and between-participant ( $r_{s}$ = 0.86) similarity values to the ideal similarity values. A possible reason behind the observable differences among the within-participant and between-participant comparison trends is that the latter can be more influenced by individual participant differences, as it directly compares the scan path sequences of multiple participants. For instance, participants requiring a larger threshold than other participants to identify (or combine) eye fixations might have resulted in the relatively stable region observed between 60 °/s to 100 °/s.

Using both computational approaches, we were able to identify and select accurate thresholds capable of adequately classifying eye fixations and saccadic movements without using the ideal visual scan paths. More specifically, the threshold values identified by both methods, 160 °/s to 280 °/s for within-participants and 180 °/s to 240 °/s for between-participants, resulted in average string similarity values over 78% similarity in the ideal trend. Furthermore, the optimal thresholds identified by both methods, 220 °/s (83.3% in ideal trend) for within-participants and 210 °/s (83.1% in ideal trend) for between-participants, outperformed the 30 °/s benchmark threshold (41.5% in the ideal trend). In addition, the threshold values identified by the proposed computational approaches closely match with the 200 °/s gaze velocity threshold identified by Komogortsev et al. (23) on a similar bullseye target-tracking task.

Thus, the proposed computational approaches expand upon prior research efforts (4), which might help researchers identify accurate thresholds without the need to manually identify the eye movements of participants, or defining them from the task or environment, which may not always be possible.

### B. Impact of threshold values on scan path sequence similarity

The results show that the impact of thresholds values on scan path sequence similarity to an ideal scan path sequence appears to follow a similar trend in other environments and tasks. More specifically, a trend where similarity values continuously increase at low thresholds until a maximum value is reached, after which the similarity values continue to decrease. Such a trend can be observed in prior research using a dot-tracking task (16) and a chess-board memory recall task (5), where the eye fixations and saccadic movements were identified using the I-DT algorithm. Our results expand upon these prior research efforts by identifying a similar trend using the I-VT algorithm on a bullseye-target tracking task.

The fact that the impact of threshold values on scan path sequence similarity appears to follow a general trend across multiple tasks and eye movement detection algorithms is important. Although future research is needed, it might be possible that between-participant and within-participant comparisons might be applicable for other eye movement detection algorithms, such as the I-DT algorithm, as well as to other tasks and environments.

### C. Limitations & future research

Although the proposed methods used the string edit algorithm compare scan path sequences to select accurate thresholds, it’s important to note that there are multiple different procedures to compare visual scan paths. In addition, the string edit algorithm contains some limitations, such as not considering the duration of eye fixations that took place when computing the similarity between scan path sequences (13). Thus, future research ought to explore applying the proposed within-participant and between-participant comparisons using scan path comparison algorithms such as MultiMatch (18; 10) or ScanMatch ( 7). These particular algorithms consider eye fixation duration when calculating the similarity between scan path sequences, which could potentially increase the performance of the proposed computational approaches.

In addition, the proposed computational approaches were applied to a simple bullseye-target tracking task. Future research should seek to investigate whether the two methods are applicable to more complex tasks that might elicit very different eye movements from participants, such as healthcare professionals inspecting an x-ray image. In such a complex task, each healthcare professional might fixate on the same AOI multiple times or even have multiple eye fixations on the same AOI. These type of behaviors might impact the performance of the proposed methods. For example, if two healthcare professionals (e.g. a novice and an expert) apply completely different visual scan paths, the underlying assumption of the between-participants method that observers apply similar visual scan paths might not be met, resulting in very low similarities across threshold values for that method.

Similarly, the proposed methods were only applied to eye movement data collected from one eye tracker – the EyeLink 1000 used by the authors of GazeBase (15). As a result, additional research is needed to evaluate whether the performance of the proposed methods is similar for eye movement data collected from eye trackers with varying sampling rates. For instance, some researchers have described how eye trackers with low sampling frequencies might not provide sufficient data for accurate saccade classification (31; 26). In turn, affecting the visual scan path sequences that can be created, used by both within-participant and between-participant comparisons to identify accurate threshold values.

Lastly, in the present study, the focus was on identifying an accurate gaze velocity threshold value for the I-VT algorithm. However, additional parameters are commonly used in the I-VT algorithm (e.g. 30), such as a minimum eye fixation duration (also used in the present study). However, other parameters exist, such as a minimum window length to calculate velocity or whether adjacent eye fixations ought to be merged based on time and angle (12). Future research should explore whether the proposed methods can be used to accurately identify threshold values for multiple parameters simultaneously.

### Ethics and Conflict of Interest

The author(s) declare(s) that the contents of the article are in agreement with the ethics described in http://biblio.unibe.ch/portale/elibrary/BOP/jemr/ethics.html and that there is no conflict of interest regarding the publication of this paper.

### Acknowledgments

We would like to thank the anonymous reviewers of this article for their thoughtful comments, comments, suggestions, and questions that helped increase the quality of the article.
